# An integrated agroforestry-bioenergy system for enhanced energy and food security in rural sub-Saharan Africa

**DOI:** 10.1007/s13280-024-02037-0

**Published:** 2024-06-01

**Authors:** Natxo García-López, Aida Bargués-Tobella, Rosa C. Goodman, Solange Uwingabire, Cecilia Sundberg, Christoffer Boman, Gert Nyberg

**Affiliations:** 1 Thermochemical Energy Conversion Laboratory, Department of Applied Physics and Electronics, Umeå University, 90187 Umeå, Sweden; 2 Department of Forest Ecology and Management, Swedish University of Agricultural Sciences (SLU), Umeå, Sweden; 3 Regional Research School in Forest Sciences (REFOREST), College of Forestry, Wildlife and Tourism, Sokoine University of Agriculture, Morogoro, Tanzania; 4 Department of Energy and Technology, Swedish University of Agricultural Sciences (SLU), Uppsala, Sweden

**Keywords:** Biochar, Cleaner cooking, Modern energy access, Restoration, Rural electrification through combined heat and power plants, Sustainable development

## Abstract

Most people in rural sub-Saharan Africa lack access to electricity and rely on traditional, inefficient, and polluting cooking solutions that have adverse impacts on both human health and the environment. Here, we propose a novel integrated agroforestry-bioenergy system that combines sustainable biomass production in sequential agroforestry systems with biomass-based cleaner cooking solutions and rural electricity production in small-scale combined heat and power plants and estimate the biophysical system outcomes. Despite conservative assumptions, we demonstrate that on-farm biomass production can cover the household’s fuelwood demand for cooking and still generate a surplus of woody biomass for electricity production via gasification. Agroforestry and biochar soil amendments should increase agricultural productivity and food security. In addition to enhanced energy security, the proposed system should also contribute to improving cooking conditions and health, enhancing soil fertility and food security, climate change mitigation, gender equality, and rural poverty reduction.

**Supplementary Information:**

The online version contains supplementary material available at 10.1007/s13280-024-02037-0.

## Introduction

Ensuring access to affordable, reliable, sustainable, and modern energy for all is an important Sustainable Development Goal (SDG 7). It is also a prerequisite and enabler for several other SDGs, including enhancing food and nutrition security, eradicating poverty, taking climate action, and improving health and well-being (IEA et al. 2023). Today, more than 700 million people lack access to electricity globally, and 2.4 billion people do not have access to clean fuels and technologies for cooking (IEA 2022b). Lack of access to clean energy often coexists with high poverty levels, gender inequalities, and food insecurity (Pachauri and Rao 2013; González-Eguino 2015). It also has major adverse effects on health and the environment, thus hindering overall sustainable development (Hosonuma et al. 2012; Bond et al. 2013; WHO 2022).

Energy poverty is unevenly distributed both between and within countries (Table 1). Sub-Saharan Africa (SSA) lags remarkably behind the rest of the world in terms of clean energy access: 52 and 82% of people lack access to electricity and clean cooking, respectively, compared to 10 and 30% of the global population. Within countries, rural populations tend to have lower access to electricity and clean cooking (World Bank 2023). Reversing these inequalities is critical to achieve the SDGs, particularly in SSA where rapid population growth is expected to exacerbate current energy and food insecurity (UN-DESA 2022).

**Table 1:** People lacking access to electricity and clean cooking, shown as total versus rural areas, respectively, globally, for Low- and Middle-Income Countries (LMIC), and sub-Saharan Africa (SSA) (World Bank 2023)

	Lack of access to electricity (% of the population)	Lack of access to clean cooking (% of the population)
Global	10	30
Global rural	17	52
LMIC	11	36
LMIC rural	19	55
SSA	52	82
SSA rural	71	94

Access to reliable and affordable electricity benefits both households and communities and is critical to drive social and economic transformation in SSA (Blimpo and Cosgrove-Davies 2019). Lighting is fundamental for education, health centers, and security (UNDESA 2014). Most African countries have pledged universal access to electricity by 2030 (IEA 2022b). In 2016, the African Development Bank launched the New Deal on Energy for Africa, with a portfolio of over US$12 billion. One of its main objectives is to stimulate the market for decentralized energy solutions such as the implementation of green mini-grids powered by, for example, biomass (AfDB 2016). Electricity from sustainably produced biomass used in clean and efficient thermochemical conversion appliances (e.g., gasification plants) could be a reliable and renewable option for rural communities (Situmorang et al. 2020).

Most people in SSA also lack access to clean cooking options and rely on traditional cooking (Table 1). Traditional cooking is typically performed on an open fire (known as a “3-stone fire”) or on other rudimentary and inefficient cookstoves fueled with solid biomass fuels (e.g., firewood or charcoal). Cooking is often done indoors with poor ventilation, resulting in high concentrations of gases and particulate matter derived from incomplete fuel combustion. These emissions are the main contributor to household air pollution, which is responsible for *ca.* 3.2 million premature deaths each year (WHO 2022). The pollutants are harmful to people directly exposed in the kitchen, primarily women and children, and contribute to climate change (as carbon dioxide, methane and black carbon) (Bond et al. 2013). In addition, inefficient stoves require large quantities of fuel. This firewood is often collected from surrounding forests and woodlands, which leads to forest and land degradation and biodiversity loss, especially in Africa (Hosonuma et al. 2012). Therefore, traditional cooking is considered an unsustainable use of bioenergy (IEA 2020). As fuelwood becomes increasingly scarce, households—typically women and girls—are forced to purchase fuels or walk ever-increasing distances to gather wood (Scheid et al. 2018). Another adaptation to fuel scarcity is to undercook (Burgess 2008) or avoid foods that require more cooking time (e.g., pulses; Sola et al. 2016), both of which contribute to undernourishment.

Several alternatives to traditional cooking have been proposed and promoted in SSA. However, most focus solely on reducing direct emissions and exposure (for human health and environmental purposes) or improving cookstove thermal efficiency (to reduce fuel consumption). Considering other sustainability aspects, such as fuel sourcing and transport or whether the energy source is renewable or affordable, is essential. For example, in SSA, liquified petroleum gas (LPG) has been widely promoted as a clean energy source (Čukić et al. 2021; IEA 2022a). While the particulate emissions from cooking with LPG are negligible, LPG is a fossil fuel that is prohibitively expensive for many (Maes and Verbist 2012) and thus not a sustainable or affordable solution. Hence, replacing traditional cooking with cleaner and more sustainable alternatives that are based on renewable energy sources is critical.

Agroforestry systems can provide sustainable biomass for energy production. Agroforestry is a set of land use systems in which trees are grown and managed on the same piece of land used for agricultural crops and/or livestock, either simultaneously or in a temporal sequence (Lundgren and Raintree 1983). Integrating trees on agricultural land can have several positive impacts, including improved crop and livestock product yields (Kuyah et al. 2016), enhanced soil health (Barrios et al. 2012; Muchane et al. 2020), reduced forest and land degradation (van Noordwijk 2019), and improved food and nutrition security (Jamnadass et al. 2013). Therefore, agroforestry can address the interconnected energy, poverty, food, water, climate, land, health, and biodiversity challenges (Kuyah et al. 2016; Rosenstock et al. 2019).

With an estimated 715 Mha of degraded land, Africa is considered the continent with the largest opportunities for Forest and Landscape Restoration (FLR) (Minnemeyer et al. 2011), and agroforestry is highlighted as a central restoration option for multi-functional landscapes (IUCN and WRI 2014; Shyamsundar et al. 2022). However, while the restoration movement has been growing rapidly during the last decade—as shown by the increasing number of restoration initiatives, such as AFR100, the Great Green Wall of the Sahel and the Sahara Initiative, or the UN Decade on Ecosystem Restoration (2021-30)—enhancing energy security through improved woodfuel supply and sustainable management of woodfuels has been a largely overlooked aspect of these initiatives (Harvey and Guariguata 2021).

It is crucial to develop sustainable solutions that address interlinked aspects of energy and food insecurity, health problems linked to traditional cooking, land degradation, and climate change. We argue that combining sustainable biomass production in agroforestry systems with modern bio-based cleaner cooking solutions and rural electricity production is a low-cost, restorative, and community-controlled solution that can simultaneously address these interlinked challenges and contribute to a transformation toward sustainability. Each of these technologies—agroforestry and bioenergy solutions—exist but, to our knowledge, have not been brought together in an integrated system. Here, we present a novel integrated agroforestry-bioenergy system and estimate its biophysical potential to provide cleaner energy for rural communities in SSA with positive food security, human health, and environmental outcomes.

## An integrated agroforestry-bioenergy system for improved energy access, food security, and human and ecosystem health

### General system description

**Fig. 1:**
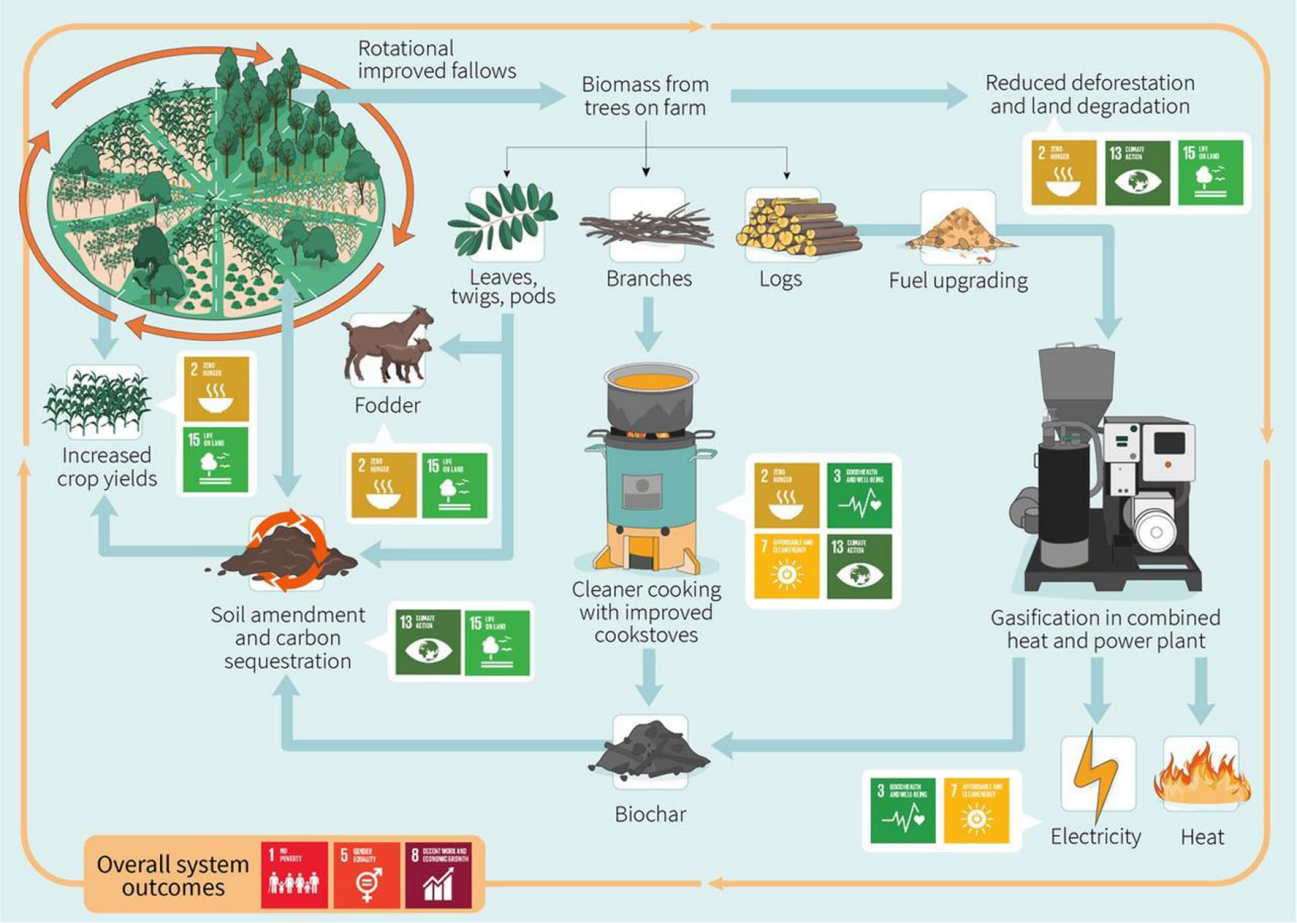
The proposed integrated agroforestry-bioenergy system and its direct contributions to SDG 7- Affordable and Clean Energy, SDG 2- Zero Hunger, SDG 3- Good Health and Well-being, SDG 15—Life on Land, SDG 13- Climate Action, SDG 5- Gender Equality, SDG 1- No Poverty and SDG 8; Decent Work and Economic Growth. Implementing cleaner cooking solutions and producing electricity and heat contribute to the achievement of SDG 7. The implementation of the system also contributes to achieving SDG 2 through improved soil health, reduced land degradation, sustainable crop intensification and livestock production, and implementation of efficient cleaner cooking solutions that enable nutritious diets. SDG 3 is supported by enhanced food and nutrition security, improved access to electricity and heat, and reduced gas and particulate emissions from cleaner cooking solutions. The sustainable production of biomass in agroforestry systems helps reduce the pressure on forests and woodlands, which, combined with improved soil health in farmlands, contributes to achieving SDG 15. Reducing land degradation and deforestation and increasing carbon sequestration in agricultural soils contribute to achieving SDG 13. Implementing this integrated system also contributes to SDG 13 by reducing emissions of particulate matter and greenhouse gases, and to SDG 5 by reducing women’s and children’s workload and the time needed to complete tasks such as collecting fuelwood or cooking food. In addition, the overall system contributes to enhancing and diversifying rural livelihoods and to achieving SDG 1 and SDG 8

The proposed integrated agroforestry-bioenergy system combines sustainable biomass production in agroforestry systems with cleaner cooking solutions and small-scale electricity production. Food production is also enhanced through improvements in soil fertility and high-quality fodder for livestock. This integrated system can provide affordable, reliable, sustainable, and modern energy for communities in rural SSA while contributing to the achievement of other SDGs (Fig. 1).

### Biomass production in agroforestry systems

Agroforestry systems are typically multi-functional (Kuyah et al. 2016) but can be designed and managed to favor the provision of specific ecosystem services. Sequential agroforestry systems—in which trees and crops are grown in rotation—are particularly suitable for fuelwood production and minimize tree-crop competition for light, water and nutrients (Sanchez 1999; Ndayambaje and Mohren 2011). Well-established sequential systems include rotational woodlots and improved fallows (Kuyah et al. 2016; Muthuri et al. 2023). Improved tree fallows are fallows in which N_2_-fixing trees such *Acacia angustissima, Acacia mangium, Calliandra calothyrsus, Gliricidia sepium, Leucaena leucocephala, Sesbania sesban,* or *Tephrosia vogelii*, are grown to accelerate the recovery of soil fertility (Sanchez 1999; Amadalo et al. 2003). Trees are harvested at the end of the fallow period, typically between 1 and 5 years, and the remaining leaves and twigs are applied as green manure to enhance soil nutrient availability. As a result, improved fallows can lead to substantial improvements in soil fertility and crop yields (Kwesiga et al. 2003; Sileshi et al. 2008).

**Fig. 2:**
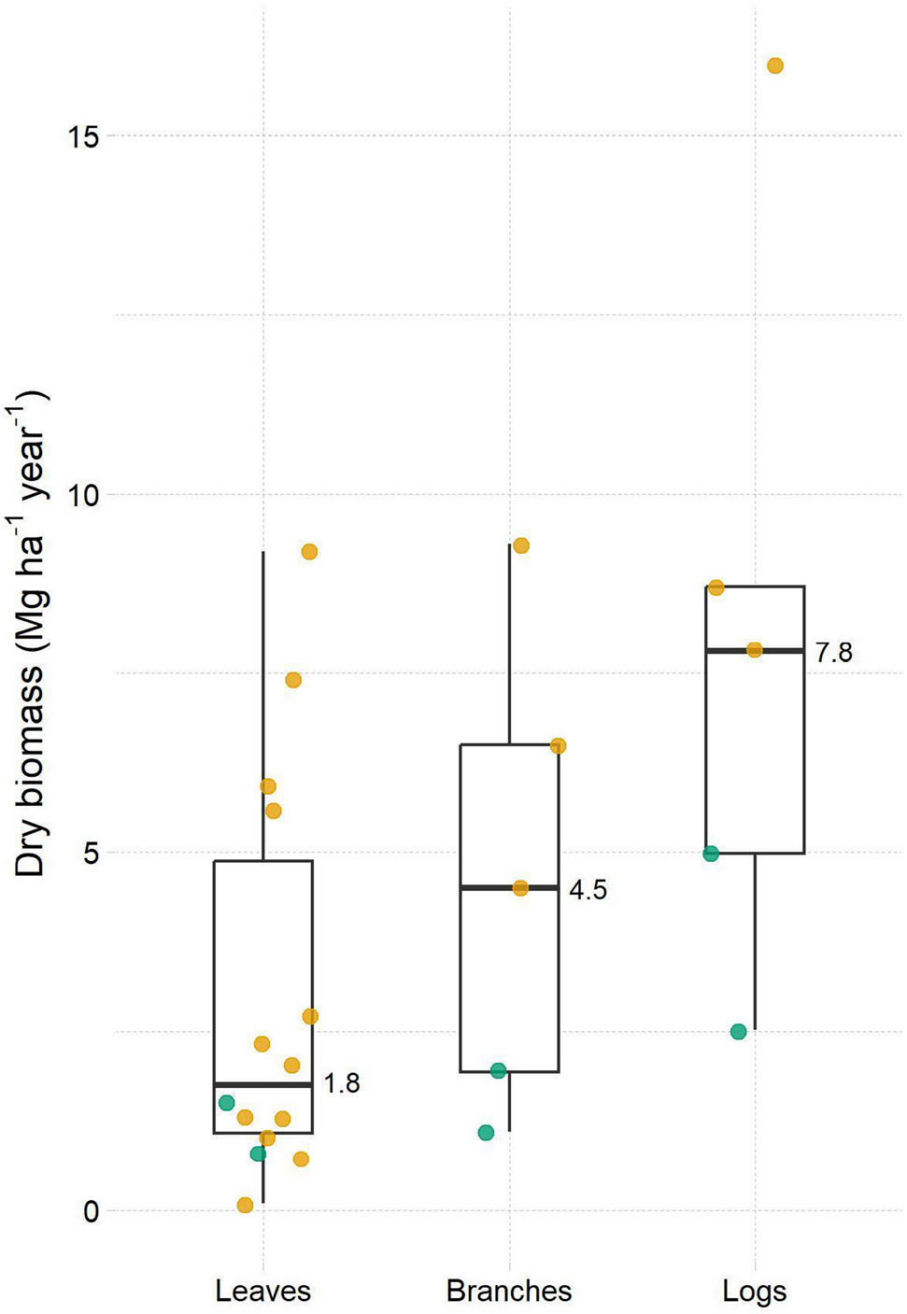
Boxplots (median, Q1 and Q3) of tree biomass production per fraction (leaves, branches, and logs) in improved fallows in sub-Saharan Africa based on primary data (green dots) and data compiled from the literature (orange dots; Table S3.1). Each data point represents the average value from multiple farms within a study

We take rotational improved fallows as the main agroforestry system example (Figure S1.1). Biomass production in improved fallows varies depending on tree species, climate, soil type, and management. We estimated tree biomass production in ten improved fallows we established in two locations in western Kenya (Appendix S2) and compiled additional data from the literature on biomass production in improved fallows across sub-Saharan Africa (Table S3.1). Total above-ground biomass production ranged between 4.3 and 27.6 Mg ha^−1^ year^−1^, with a median of 8.7 Mg ha^−1^ year^−1^ (Table S3.1). When considering biomass production for different tree fractions, the median values were 1.8, 4.5, and 7.8 Mg ha^−1^ year^−1^ for leaves, branches, and logs, respectively (Fig. 2, Table S3.1).

To estimate the tree biomass production at the farm level, we assume that each farm is 1 ha and allocates 20% of its area (0.2 ha) to biomass production in rotational improved fallows (Figure S1.1). We assume a fallow period of two years (see Table S4.1 for a complete list of assumptions). Each year, 0.1 ha of cropland is set into fallow and planted with N_2_-fixing trees. After two years, the trees in the improved fallow are harvested, and crops are grown again. The first two years following the system implementation constitute the establishing phase, where no trees can be harvested. After this phase, 0.1 ha of land under improved fallow is harvested per farm each year. We take the median biomass production values obtained for each fraction to estimate the tree biomass production per farm and year for leaves, branches, and logs. This results in 360, 900, and 1560 kg of leaves, branches, and logs, respectively (expressed as dry basis), per farm each year starting at the beginning of the third year.

### Biomass fractions and utilization

Each of the three biomass fractions—(i) leaves, twigs and pods; (ii) branches; and (iii) logs–can be utilized distinctly within this system. Leaves, twigs, and pods are most suitable for soil amendment and as livestock fodder, thereby improving overall agricultural productivity. Branches are optimal as fuel for improved cookstoves. Larger woody biomass (i.e., logs) can be upgraded into woodchips, which can be used for electricity production through gasification in small-scale combined heat and power (CHP) plants. In addition to electricity, heat and biochar are valuable co-products obtained through gasification. The residual heat generated during the gasification process can also be used, for example, to dry crops or biomass. Biochar can be used as soil amendment. Additionally, growing nitrogen-fixing trees in improved fallows adds organic matter and nitrogen to the soil, which improves soil fertility and yields from subsequent crops.

### Biomass-based cleaner cooking solutions

Cleaner cooking solutions—defined here as biomass-based cooking solutions with lower fuel consumption and better emission performance than the traditional 3-stone fire—can be broadly divided into improved and advanced cookstoves (Kshirsagar and Kalamkar 2014). Advanced cookstoves use upgraded fuels (typically pellets) and forced air supply. However, in most places in rural SSA today, advanced cookstoves are not a feasible option given the challenges related to stove and fuel affordability and availability (Mulenga and Roos 2021). Hence, in this perspective piece, we focus on improved cookstoves such as rocket stoves and natural draft gasifiers. These cookstoves are generally fueled with unprocessed biomass, although some use woodchips, carbonized biomass such as charcoal (most commonly produced in traditional and inefficient earth kilns), or briquettes made from carbonized biomass. In this study, we estimate the system outcomes considering improved cookstoves fuelled with unprocessed biomass (i.e., dried branches). Improved cookstoves can be constructed according to many different designs and materials but are, in principle, relatively simple and robust combustion units (Urmee and Gyamfi 2014). Given their improved combustion and thermal efficiency, improved cookstoves have an overall better emission performance per unit fuel than a 3-stone fire and lower total gas and particulate emissions per cooking event (MacCarty et al. 2010; Jetter et al. 2012). It has been shown that particulate matter emissions from improved cookstoves can be less than one-third of those resulting from using the 3-stone fire to complete the standard Water Boiling Test (MacCarty et al. 2010). Because of reduced CO, fine particle, and polycyclic aromatic hydrocarbons (PAH) emissions, improved cookstoves also benefit health (Pratiti et al. 2020).

**Fig. 3:**
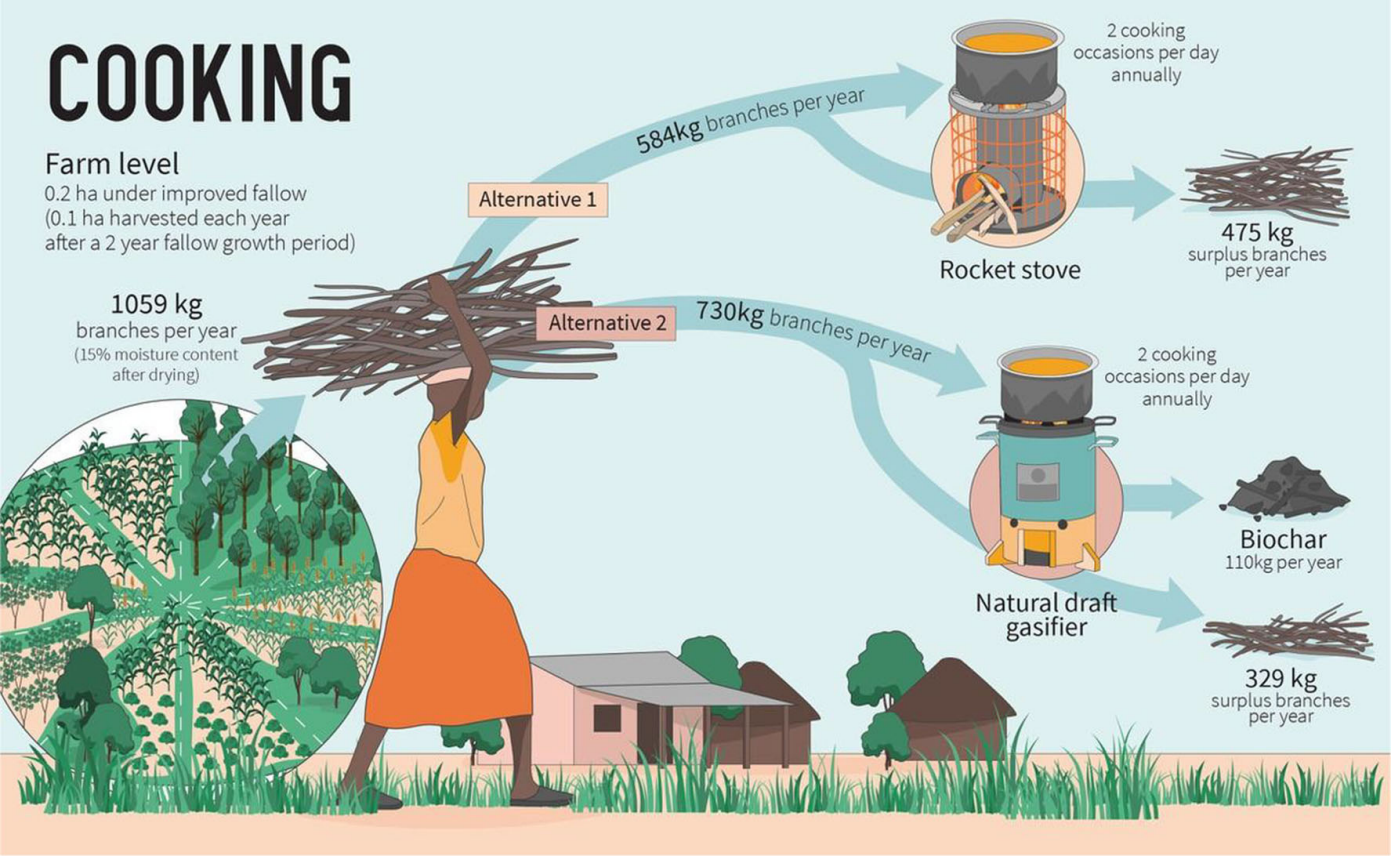
Branches produced in improved fallows can be used for cooking in two different improved cookstove technologies, i.e., rocket stoves (alternative 1) or natural draft gasifiers (alternative 2). Two cooking occasions per day annually require an estimated 584 kg of branches when cooking with rocket stoves, while natural draft gasifiers would consume 730 kg. The produced biomass (1059 kg of branches per year) would cover these cooking needs, and there would still be a surplus of fuel. Natural draft gasifiers also produce biochar

We estimate the number of cooking events per household and year that could be performed with biomass from improved fallows in their own farmland (Fig. 3). We assume that the households cook with improved cookstoves exclusively. Assuming a production of branches of 4.5 Mg dry mass ha^−1^ year^−1^ (Fig. 2, Table S3.1) and a fuel moisture content of 15% during utilization, each household would have access to 1059 kg of branches per year.

Fuel consumption in improved cookstoves varies among different technologies. In a lab study comparing fuel consumption and emissions performance for 50 different cookstoves, fuel consumption for rocket stoves was 608–1282 g (median 757 g) to complete the water boiling test, which consists of boiling 5 L of water and keeping them simmering for 45 min; for natural draft gasifiers, fuel consumption was 741–1234 g (median 961 g; MacCarty et al. 2010). However, fuel consumption for natural draft gasifiers was found to be slightly higher in a field study: 767–1944 g (median 1119 g) when cooking different food recipes (Gitau et al. 2019). Based on these values, we assume a fuel consumption of 800 and 1000 g for rocket stoves and natural draft gasifiers, respectively. If each household produces 1059 kg of branches per year and cooks twice a day, they would still have a fuel surplus of 475 and 329 kg a year for rocket stoves and natural draft gasifiers, respectively (Fig. 3). Natural draft gasifiers also produce biochar. Based on biochar production values reported by Gitau et al. (2019), we assume a biochar production of 15% of the fuel mass. Thus, 150 g of biochar is produced per cooking occasion and 110 kg year^−1^ is produced by each household using natural draft gasifiers (Fig. 3).

### Small-scale electricity and heat production through biomass gasification

Electricity can be generated from biomass through thermochemical energy conversion in different technological solutions, for example combining biomass combustion in a boiler with a steam turbine or in systems combining biomass gasification with a power generator run by an internal combustion engine. Boilers combined with steam turbines are the most commonly used technology in industrial-scale plants (Dong et al. 2009). However, biomass gasification (e.g., in fixed-bed appliances) combined with power generators is a well-established technology (Allesina and Pedrazzi 2021) and the most suitable option for small-scale electricity production in rural areas (Situmorang et al. 2020).

From a thermochemical conversion point of view, biomass is composed of volatile components, fixed carbon, and ash-forming components. Gasification is a thermochemical energy conversion process through which biomass is converted (partially combusted) into an energy-rich gas stream and solid fractions (ash and char). Gasification is an intermediate process between pyrolysis (that occurs in the absence, or nearly absence, of oxygen) and complete combustion (unlimited access to oxygen) (Brown 2019). Different proportions of volatile components (gases), fixed carbon (biochar), and ash are produced depending on the gasification process conditions, e.g., temperature, residence time, and air supply (oxygen availability). When the primary purpose of gasification is to produce electricity, the production of an energy-rich gas is maximized (Mishra and Upadhyay 2021).

**Fig. 4:**
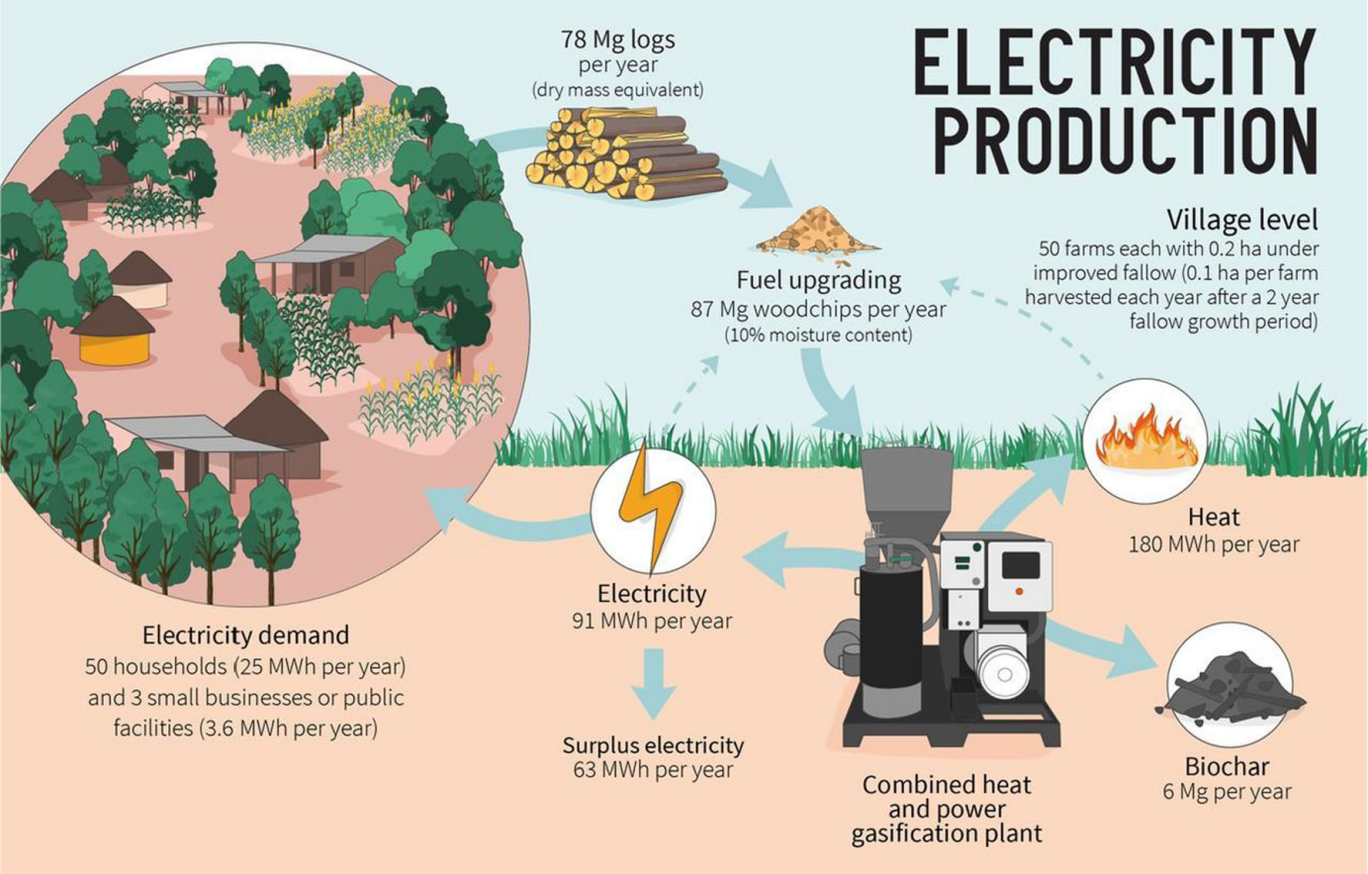
The logs produced in improved fallows can be used to generate electricity in small-scale combined heat and power (CHP) gasification plants that also produce heat and biochar. An estimated 78 Mg of logs (dry mass equivalent) are produced annually at the village level (assuming 50 farms per village, each with 0.2 ha under improved fallows). Fresh logs are upgraded (dried to 10% moisture content and chipped) and used to produce electricity in the CHP plant. The produced electricity covers the annual village electricity demand and there is still a surplus. Part of this surplus can be used during fuel upgrading. Besides electricity, heat, and biochar are also produced. The produced heat can be used to dry the biomass

In the proposed integrated agroforestry-bioenergy system, logs are upgraded into woodchips and used to produce electricity in small-scale combined heat and power (CHP) plants. This is a commercially available technology (Allesina and Pedrazzi 2021; Mishra and Upadhyay 2021) consisting of a fixed-bed biomass gasification reactor combined with an internal combustion engine and a power generator (Fig. 4). We estimate the amount of electricity that could be produced at the village level if 50 households grow trees in improved fallows (0.2 ha each) and supply the logs to the gasification plant. Assuming a production of logs of 7.8 Mg dry mass ha^−1^ year^−1^ (Fig. 2, Table S3.1) and a fuel moisture content of 10% during utilization, the annual amount of available logs biomass at the village level would total 87 Mg (Fig. 4). We assume a lower moisture content in the logs, compared to branches, because there is access to biomass dryers that utilize heat from the CHP plant.

Several manufacturers offer small-scale CHP plants based on biomass gasification, and we have compiled technical specifications for 16 CHP plants with an electric power ≤ 50 kW from 10 different manufacturers (Table S5.1). We base our calculations on a CHP plant with an electric and thermal power of 40 and 79 kW, respectively, a fuel consumption of 38 kg h^−1^ wet-basis (ranging 18–60), and a biochar production of 2.7 kg h^−1^, which correspond to average values of the compiled commercially available plants (Table S5.1).

We assume that energy consumption per household is 500 kWh year^−1^, which corresponds to the first level of the electricity ladder established in the Africa Energy Outlook for urban households (IEA 2022a). This is twice as much as rural households would use when they first gain access to electricity (250 kWh year^−1^; IEA 2022a), which offers a conservative estimate of the number of households that the proposed system can power. We also assume that each village has three small businesses or public facilities, each consuming 1.2 MWh year^−1^, which is in line with the reported figures by Muhwezi et al. (2021). Retail shops, schools, health care centers, restaurants, and welding workshops are examples of small businesses and public facilities typically found in rural areas. Thus, a village of 50 households and three small businesses or public services would have an annual electricity need of 28.6 MWh.

From the 87 Mg of processed logs, 91.2 MWh of electricity are generated in the CHP plant, which would cover the village’s electricity demand and still result in a surplus of 62.6 MWh electricity annually (i.e., the equivalent to the demand of two additional villages of the same characteristics; Fig. 4). Additionally, 180 MWh of heat and 6 Mg biochar would also be produced in the CHP plant. Part of the electricity surplus and the heat could be used for fuel drying and upgrading, such as wood chipping at the CHP plant (Fig. 4). The heat could also be used to dry crops, water potabilization, etc.

Based on our assumptions, the CHP plant would be able to operate for about 7 h per day, 6 days a week. If it is important to increase the return on investment to make the system more attractive to funders and investors, the CHP plant could be run closer to full-time. If the plant operates for 10 h a day, 6 days per week, it would take about 70 farms to supply enough biomass.

### Biochar production and utilization

Biochar is the product of charring or pyrolyzing biomass in temperatures above about 350˚C (Lehmann and Joseph 2015). In the proposed system, biochar is obtained as a co-product from both household cooking in natural draft gasifier stoves and electricity production in CHP plants. Biochar can then be used as soil amendment. This provides sequestration of biogenic carbon in the soil and makes biochar a long-term CO_2_ removal technology (Lehmann et al. 2021; Schmidt et al. 2021; IPCC 2022). Biochar can improve soil fertility and crop yields, and the effects last for several years (Kätterer et al. 2019, 2022; Dai et al. 2020; Ye et al. 2020). Results from a global meta-analysis of the effect of biochar addition on crop yield indicated a 15% average increase in yields in the tropics, with the highest increase at a biochar application rate of 5–10 Mg ha^−1^ (Ye et al. 2020). Kätterer et al. (2022) show substantially higher yield increases of maize already at application rates of 1 Mg biochar ha^−1^ on farmers’ fields in Kenya.

Based on our estimates, our agroforestry-bioenergy system could generate between 122 and 232 kg of biochar per household and year (6.1 Mg year^−1^ per village of 50 households from the CHP gasification plant, and an additional 110 kg year^−1^ per household if cooking with natural draft gasifiers). Biochar from the gasification plant could be marketed as a product for soil amendment. Considering the relatively low biochar production at the household level (max. 232 kg year^−1^ in the case when households are using the natural draft gasifiers), it would be beneficial to concentrate biochar amendments to higher-value vegetables or crops (in nutritional or economic terms) during the first years following the system establishment. In the forthcoming years, biochar amendments could be applied over larger areas as a substantial cumulative effect of biochar application over time can be expected (Kätterer et al. 2019; Ye et al. 2020). The combination of biochar application to the soil, improved fallows that use N_2_-fixing trees, and the addition of manure can have significant positive effects on soil fertility and agricultural productivity, with potential beneficial impacts on nutrition and income. There might also be synergies when using these together, for instance, through reduced soil nutrient losses via leaching (Lehmann and Joseph 2015).

### Overall system outcomes

The proposed system can contribute to multiple prongs of sustainable development (Fig. 1). In addition to the direct contributions to the SDGs described earlier, there are also overall system outcomes—including poverty alleviation, economic growth, and gender equality – that result from the combination of more specific outcomes and their synergies.

In SSA, 556 million people live in multidimensional poverty, the vast majority of whom (82%) are in rural areas (UNDP and OPHI 2021). Our integrated agroforestry-bioenergy system explicitly targets these rural communities and contributes to improved well-being and poverty reduction primarily through two dimensions of poverty: health and standard of living. Increased energy security through improved access to electricity and cleaner cooking raises household living standards, while cleaner cooking and enhanced food and nutrition security improve health (FAO et al. 2023; IEA et al. 2023). The system also contributes to monetary poverty reduction through income diversification, enhanced employment opportunities, and increased profits for farmers. In addition to poverty alleviation, the proposed system contributes to building a circular and sustainable bioeconomy in rural areas, enhancing sustainable economic growth and productive employment (Openshaw 2010).

Women and children primarily bear the costs of energy poverty, as they are generally responsible for fuelwood collection and cooking activities (Clancy et al. 2003; Njenga et al. 2021). These costs include labor, time, and related opportunity costs, as well as adverse health impacts from exposure to indoor air pollution (Ali et al. 2021). Implementing our integrated agroforestry-bioenergy system would reduce the burden on women to collect firewood from forests or woodlands through an on-farm sustainable supply of biomass fuels; reduce indoor air pollution through cleaner cooking solutions; and free up time and energy to spend on other pursuits, such as education or business activities. Enhancing gender equality will also require taking measures to ensure women’s access to financial services and markets and control over income and benefits from the system implementation—e.g., from selling woodfuels—and avoid co-optation by men.

## System implementation and upscaling

Realizing the full potential of the proposed integrated agroforestry-bioenergy system requires adopting its different underlying technologies. We discuss potential barriers to implementation and ways to overcome them.

The main barrier to adopting improved fallows by small-scale farmers in SSA is that farmers cannot afford to take land out of agricultural production, even when crop harvest is very low (Franzel 1999; Meijer et al. 2015). This is a serious concern, especially when farm sizes are small, and any transition will need to manage this carefully. It is well-known that the provision of direct economic benefits resulting from agroforestry is a key factor in determining adoption potential (Meijer et al. 2015). We envisage that the woodfuel market will drive the transition to this agroforestry-bioenergy system. The sale of stem wood produced in the improved fallows to the CHP plants should compensate for the temporary loss of cropland during the system establishing phase and create an economic incentive for farmers to use improved fallows and other agroforestry methods. In addition, farmers will benefit from increased soil fertility, access to animal fodder, and fuelwood availability for household cooking. However, some up-front payments may be needed as many small-scale farmers do not have planning horizons beyond the next growing season (Ajayi et al. 2003; Pannell et al. 2014). For example, the CHP plants could pay in advance for the purchase of stem wood. Here, we have assumed a 2-year fallow period, which is a conservative estimate. Fast-growing species could produce enough biomass in less than two years (Table S3.1), thus reducing this barrier.

In Rwanda, one of the most densely populated countries in SSA, the average farm size is 0.72 ha (NISR and MINAGRI 2007). Similarly, Marinus et al. (2022) showed that median farm size in other regions in the East African highlands ranged between 0.8 and 1.8 ha. In less-densely populated regions across SSA, farms over 5 ha are more common (Jayne et al. 2022). Thus, allocating 0.2 ha for biomass production is substantial but not unreasonable. Exact sizes and design will need to be adjusted based on context to fit the needs of the farmers and communities. Furthermore, as soil fertility improves, crop yields can increase by 50–200% (Kwesiga et al. 2003; Hall et al. 2006; Sileshi et al. 2008), which provides a strong incentive for farmers to use improved fallows (Franzel 1999).

The implementation of cleaner cooking technologies has proved challenging (Boudewijns et al. 2022). The high up-front cost of new stoves and limited access to credit is the primary barrier to adoption, even though firewood saving due to higher cookstove efficiency typically translates into high returns on investment (Bensch et al. 2015; Boudewijns et al. 2022). Addressing these affordability constraints, for instance, through lending schemes or subsidies (Rehfuess et al. 2014; ESMAP 2021), is critical. Other factors inhibiting wider uptake of improved cookstoves include knowledge and beliefs concerning the innovation and cookstove compatibility with the local context, e.g., cooking needs, local practices, fuel type and availability, season, geography and culture (Boudewijns et al. 2022). Thus, central strategies to facilitate implementation include considering each individual’s attitudes toward improved cookstoves, ensuring compatibility between the improved cookstove technology and the local context, ensuring that there is a perceived advantage of improved cookstoves compared to traditional cooking, and facilitating access to information and knowledge about these stoves and how to use them (Boudewijns et al. 2022).

Rural electrification will also require considerable investment in both the CHP plants and the actual mini-grids. However, the clear alignment with national and international goals and pledges to increase access to electricity in rural areas might generate opportunities to finance the transition, e.g., through subsidies and grants. Promoting productive, income-generating uses of electricity (e.g. businesses and industry) is also important to finance the construction and maintenance of mini-grids (AfDB 2016). The main constraint to mini-grid implementation and upscaling in Africa is the current policy and regulatory framework gaps, including aspects related to tariffs and licensing (AfDB 2016). Addressing these gaps is essential. Some African countries, such as Mali, Senegal, Sierra Leone, Kenya, the United Republic of Tanzania, have already introduced fiscal incentives supporting renewable energy solutions (IRENA and AfDB 2022). The lack of capacity of key stakeholders across different levels of the mini-grid market is another barrier to consider (AfDB 2016). Therefore public institutions, private companies, and local stakeholders will need to develop their capacity and technical skills in order to construct, administrate, operate, and maintain these rural, biomass-based mini-grids (Bhattacharyya and Palit 2016). Further, ensuring a reliable fuel supply is critical for the proper functioning of the CHP plant and for anyone to invest in its development. Hence, logistic aspects such as harvesting, transportation, storage, and fuel upgrading (e.g., wood chipping) will require careful planning, management, and financing. The organization of the woody biomass supply chain to the CHP plants needs to be context-specific, e.g., it may be through farmers’ cooperatives or outgrower schemes. However, this will have to be agreed upon at the local level.

A well-functioning and readily available fuelwood market and expanding rural electrification will help drive this system’s implementation and upscaling. Abundant and reliable fuelwood becomes an incentive to establish CHP plants, while the possibility of selling surplus fuelwood to these plants incentivizes farmers to produce fuelwood. Additionally, improved fallows and biochar amendments increase crop productivity and, thus, food security. The positive feedback between enhanced soil fertility and improved yields resulting from biochar application can lead to a self-amplifying feedback loop that reinforces the system. Moreover, improved efficiencies in household cooking will enable the sale of larger proportions of household fuelwood. Rural electrification may incentivize investments and job creation, drive overall development, and reduce fossil fuel dependency.

The proposed system creates clear opportunities for funding via carbon finance mechanisms. The use of agroforestry, especially improved fallows, increases above- and below-ground carbon sequestration (11.3 Mg C ha^−1^ yr^−1^ and 1.9 Mg C ha^−1^ yr^−1^, respectively) (Feliciano et al. 2018). The feedstock for biochar is photosynthetically fixed CO_2_; hence, applying biochar to agricultural soil is essentially a carbon dioxide removal technology (Lehmann et al. 2021; Schmidt et al. 2021; IPCC 2022). In addition, replacing traditional cooking with improved cookstoves can mitigate carbon-equivalent emissions of climate-forcing agents (Smith and Haigler 2008; Grieshop et al. 2011), although improved cookstove programs in real-life settings might not always achieve expected carbon reductions (e.g., Aung et al. 2016). In total, there are net negative carbon emissions when biochar from cleaner cooking with renewable biomass (e.g., agroforestry trees) is used as soil amendment (Sundberg et al. 2020). Carbon financing could thus be an additional incentive to drive the transition to a sustainable agroforestry-bioenergy system or provide initial financing (Usmani et al. 2017).

If the proposed integrated agroforestry-bioenergy system is not implemented in its totality, there are still a range of intermediate stages that would provide a number of benefits. For example, just having an on-farm biomass production in improved fallows and transitioning to cleaner cookstoves instead of relying on traditional cooking could bring substantial benefits at the household level. It is also possible to fuel improved cookstoves with prunings from agroforestry trees such as fruit trees if improved fallows are not implemented. The use of biochar as a soil amendment may need training and adaptation to local crops and soils. The system could also be implemented at the interface between rural and peri-urban areas. Farmers in rural areas could grow the trees in agroforestry systems, and supply stem wood to CHP plants located in peri-urban areas with higher population density and electricity demand. This approach would still lead to enhanced soil fertility, reduced forest and woodland degradation, and provide households with sustainably produced biomass for cleaner cooking solutions, as well as animal fodder. In addition, farmers would generate income by trading stem wood for gasification in peri-urban areas.

For farmers to adopt this system, they will need a reliable market for fuelwood, but for anyone to invest in a bioenergy system, they will need a reliable supply of fuelwood. We hope that building integrated, self-sustaining, and economically sound systems will become the new kind of development, where finance is only needed for initial investments in equipment (e.g., improved cookstoves and a bioenergy plant), capacity building, and enabling the transition during the first few years. Though the proposed system has only been ‘tested’ in theory, it shows that with very achievable growth rates, biomass production from small patches of improved fallows can meet annual cooking needs plus a substantial surplus for off-farm income and rural electricity generation. With improved agroforestry management through practices such as thinning and pruning, biomass production could be even higher.

The proposed integrated agroforestry-bioenergy system will need to be adapted to local ecological and socio-economic contexts. Such adaptations can occur at different levels. At the farm and household levels, this might include choices about tree species used in improved fallows, farmland area under biomass production, duration of fallows, cookstove technology/design used, and biochar use. At the village or community level, it could be about the organization of the supply chain to the CHP plants. Co-designing these adaptations together with local communities and other stakeholders is paramount to ensuring the successful adoption of the system and sustainable system outcomes, as well as to identifying system trade-offs (Coe et al. 2014; Dumont et al. 2019; Boudewijns et al. 2022).

In this piece, we have estimated the biophysical system outcomes and briefly discussed potential implementation barriers and ways to overcome them. However, there are other critical aspects related to the system implementation—notably governance, financial, and gender aspects. Analyzing these aspects is outside the scope of this piece, but we hope that follow-up studies will investigate them in detail.

## Concluding remarks

We propose a novel integrated system that links restorative agroforestry, sustainable biomass production for household cooking and enhanced soil fertility, cleaner and more efficient cooking solutions, an organized wood market for small-scale farmers, and electricity generation for rural communities. Even with conservative assumptions, we show that the proposed system offers great potential to provide cleaner energy for rural communities in SSA and generates multiple positive outcomes. It contributes to ensuring access to reliable, sustainable, and modern energy for rural communities in SSA, and to several other sustainable development goals. Farmers within the system could gain an additional revenue stream through the sale of excess wood while also benefiting from enhanced soil fertility and food production. Households would need less fuel for cooking and benefit from a healthier cooking environment through reduced exposure to air pollution. Moreover, rural electrification could drive general development on village, regional, and national scales. Globally, we will all benefit from reduced emissions and environmental degradation, as well as increased carbon sequestration and food production. The integration of the different technologies—improved fallows, improved cookstoves, CHP gasification plants, biochar—and the synergies that the system generates will contribute to overcoming well-known implementation barriers of the separate system components. The proposed integrated agroforestry-bioenergy system addresses the sustainable management and sourcing of woodfuels, aspects that have been largely overlooked in initiatives related to Forest and Landscape Restoration or aiming at enhancing access to affordable, reliable, and modern energy services. Accelerating the use of integrated solutions like this will also require more integrated policy approaches.

## Supplementary Information

Below is the link to the electronic supplementary material.

Supplementary file 1 (PDF 689 KB)
