# Supplementary material for: An integrated agroforestry-bioenergy system for enhanced energy and food security in rural sub-Saharan Africa
Source: Ambio. 2024 Jun 1;53(10):1492–504. doi: 10.1007/s13280-024-02037-0 (PMC11383902; doi:10.1007/s13280-024-02037-0)
Supplement: Supplementary file 1 — (PDF 689 KB) [file 13280_2024_2037_MOESM1_ESM.pdf]

Ambio

Supplementary Information

This supplementary information has not been peer reviewed.

Title: **An integrated agroforestry-bioenergy system for enhanced energy and food security in rural sub-Saharan Africa**

Appendix S1. Rotational improved fallows

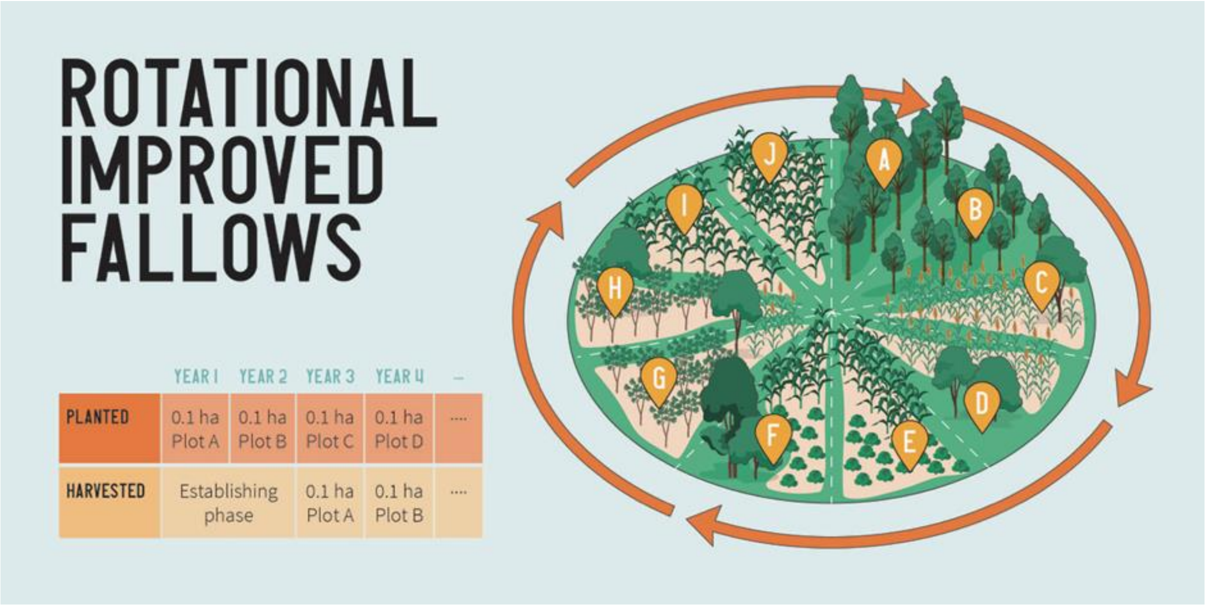

Figure S1.1. Improved fallows are sequential agroforestry systems in which trees and crops are grown in rotation. We assume that each farm is 1 ha in total and that 0.2 ha are dedicated to tree biomass production under improved fallows. We consider a fallow period of 2 years. After a 2-year establishing phase, 0.1 ha are harvested annually, starting at the beginning of year 3. The illustration represents a snapshot of a farm at the end of year 2, showing 2-year-old trees in plot A and 1-year-old trees in plot B. The remaining plots are under cultivation with different crop types. At the beginning of year 3, the trees in plot A will be harvested, and the plot will go under cultivation, while new trees will be planted in plot C.

Appendix S2. Estimation of tree biomass production in improved fallows

In addition to the data on tree biomass production in improved fallows gathered from the literature, we collected own data from two sites in western Kenya. *Sesbania sesban* fallows were established in ten farms - four in Kitalale (Trans-Nzoia county) and six in Siaya (Siaya county) - in April 2017 (Table S2.1). The area under improved fallow varied between 0.03 and 0.13 ha depending on the farm characteristics and the farmer's preferences.

Table S2.1. Location and area of the 10 Sesbania sesban improved fallows in western Kenya where we estimated tree biomass production.

Farm id	Latitude	Longitude	Area (ha)
Siaya 1	0.019898	34.40516	0.042
Siaya 2	0.000958	34.40116	0.030
Siaya 3	-0.00095	34.39686	0.050
Siaya 4	-0.0036	34.41544	0.098
Siaya 5	0.003199	34.43329	0.046
Siaya 6	0.0017	34.41056	0.130
Kitalale 1	1.007727	34.91746	0.124
Kitalale 2	1.02044	34.91838	0.035
Kitalale 3	0.998358	34.92361	0.042
Kitalale 4	0.999042	34.92389	0.050

A tree inventory was conducted in December 2019 in the ten improved fallows. All trees above 10 mm Diameter at Breast Height (DBH) were labelled and measured for DBH using a caliper. Based on the DBH distribution (Figure S2.1), we established four DBH classes (10 - 25 mm; 25 - 50 mm; 50 - 75 mm and >75 mm). For each class, 10 trees were randomly selected for harvest, totaling 40 trees.

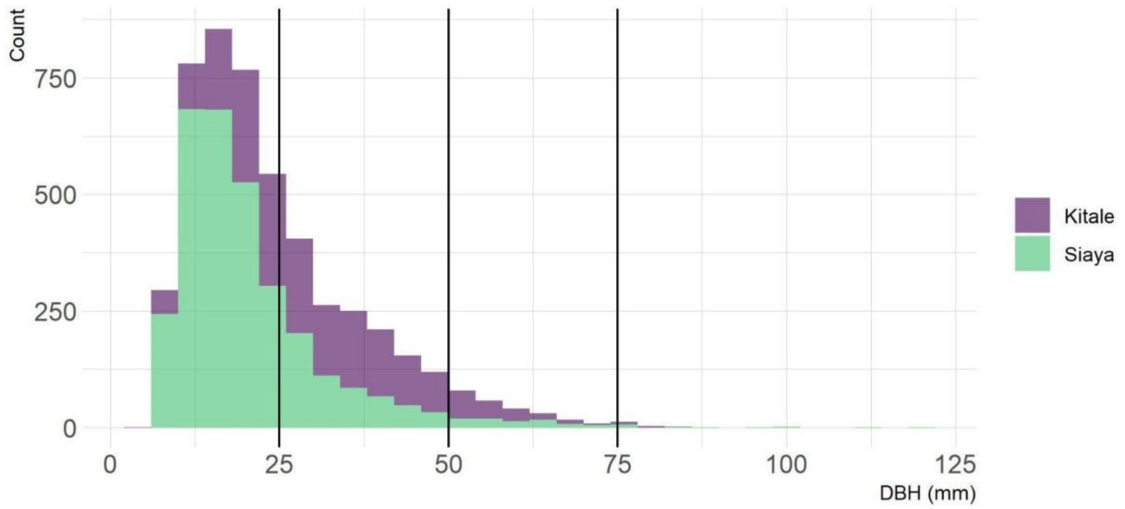

Figure S2.1. Diameter at breast-heigh (DBH) distribution of *Sesbania sesban* trees in the ten study improved fallows in Kitalale and Siaya sites, western Kenya (fallow period of 34 months). The vertical lines indicate the DBH class thresholds.

The 40 selected trees were harvested in February 2020, i.e., 34 months after the woodlots were established. Harvested trees were divided into three fractions: Leaves, pods and twigs below 5 mm in diameter; branches (between 5 and 20 mm in diameter); and logs (above 20 mm in diameter). For each harvested tree, we recorded the total fresh mass of each fraction and collected two sub-samples from each fraction (n = 240 sub-samples in total) to determine the moisture content. The sub-samples were dried at 105 °C until no significant decrease in mass was detected, and the final dry mass was recorded. The moisture content for each sub-sample was calculated as a percentage of fresh mass and averaged per tree and fraction. Finally, the dry mass for each harvested tree and fraction was estimated based on its total fresh mass and mean moisture content.

Allometric models were developed to estimate the tree biomass for each fraction in the ten improved fallows based on the DBH (cm) and dry mass (kg) data from the 40 harvested trees. Due to non-linearities in the data, we performed a log-log transformation of the DBH and dry mass data. We then fitted least-square linear regression models to the transformed datasets (ln (dry biomass) = $\beta_0 + \beta_1$ ln (DBH)), one model for each fraction, using the lm function within the stats package in R. The estimates of the regression coefficients, their associated standard error and p-value, and the $R^{2,}$ for each model, are presented in Table S2.2. Plots of residuals were used to visually assess that model assumptions of normality, linearity, and homoscedasticity were not violated. We then used these regression models to predict the dry biomass of the different fractions for all individual trees in the 10 improved fallows based on their DBH. Model predictions computed on the logarithmic scale must be transformed to the original scale (kg). However, a simple exponential-based transformation generates bias (Finney 1941), and applying correction factors is often recommended to correct this bias (Clifford et al. 2013). Here, we used the MM correction factor (Shen and Zhu 2008), as recommended by Clifford et al. (2013) when predicting the biomass of new trees. The total predicted biomass per fraction for each of the ten improved fallows is shown in Table S2.3.

Table S2.2. Regression model summaries for the three models, one for each of the defined tree fractions (leaves, twigs and pods; branches; and logs).

**Regression model**	**β_0**			**β_1**			**R^2**
	**Estimate**	**Std. error**	**p-value**	**Estimate**	**Std. error**	**p-value**	
ln (dry mass leaves) = $\beta_0 + \beta_1$ ln (DBH)	-3.6	0.14	<0.001	2.3	0.09	<0.001	0.94
ln (dry mass branches) = $\beta_0 + \beta_1$ ln (DBH)	-2.6	0.19	<0.001	1.7	0.13	<0.001	0.83
ln (dry mass logs) = $\beta_0 + \beta_1$ ln (DBH)	-3.2	0.21	<0.001	2.8	0.14	<0.001	0.92

Table S2.3. Predicted total dry biomass (kg) per tree fraction for each improved fallow.

Farm id	Leaves, pods and twigs (kg)	Branches (kg)	Logs (kg)
Siaya 1	154	197	544
Siaya 2	67	107	191
Siaya 3	69	87	251
Siaya 4	255	418	708
Siaya 5	133	167	470
Siaya 6	48	97	107
Kitalale 1	352	457	1173
Kitalale 2	245	292	872
Kitalale 3	147	213	446
Kitalale 4	176	240	564

Appendix S3. Compiled data on biomass production in improved fallows

Table S3.1. Tree biomass production from selected species in improved fallows across sub-Saharan Africa and median, Q1, and Q3 values. Mass is expressed on a dry basis. The tree species, location, Köppen-Geiger climatic zone (Kottek et al. 2006), mean annual precipitation, dominant soil type (order) according to the USDA soil classification system (USDA–NRCS 1999), fallow period, tree density, and source are indicated .

Tree species	Country	Köppen-Geiger climate classification	Mean annual precipitation (mm)	Dominant soil type	Fallow period (months)	Tree density (trees/ha)	Logs	Branches	Total aboveground woody biomass	Leaves	Total aboveground biomass	Sample size (Number of sampled improved fallows)	Source
							(Mg ha^{-1} year^{-1})						
Sesbania sesban	Kenya	Cwb - Subtropical highland climate	900	Mollisol and Alfisol	22	10000	8.7	6.5	15.2	2	17.1	4	(Ståhl et al. 2002)
Sesbania sesban (managed for pole production)	Kenya				22		16	9.3	25.3	2.3	27.6	4	(Ståhl et al. 2002)
Calliandra calothyrsus	Kenya				22		7.8	4.5	12.3	1	13.4	4	(Ståhl et al. 2002)
Sesbania sesban	Zimbabwe	Cwb - Subtropical highland climate	750	Alfisol	12	10000	-	-	1.4	7.4	8.8	3	(Mafongoya and Dzowela 1999)
Sesbania sesban	Zimbabwe				24		-	-	3.1	5.6	8.6	3	(Mafongoya and Dzowela 1999)
Sesbania sesban	Zimbabwe				36		-	-	6.2	1.3	7.5	3	(Mafongoya and Dzowela 1999)
Acacia angustissima	Zimbabwe				12		-	-	2.5	2.7	5.2	3	(Mafongoya and Dzowela 1999)
Acacia angustissima	Zimbabwe				24		-	-	10.9	9.2	20.1	3	(Mafongoya and Dzowela 1999)
Acacia angustissima	Zimbabwe				36		-	-	7	5.9	12.9	3	(Mafongoya and Dzowela 1999)
Acacia angustissima	Zimbabwe	Cfb - Subtropical highland climate	750	Alfisol	24	10000	-	-	7.6	0.7	8.3	9	(Chikowo et al. 2004)

Sesbania sesban	Zimbabwe				24		-	-	4.5	1.3	5.8	9	(Chikowo et al. 2004)
Sesbania sesban	Zambia	Cwa - Monsoon-influenced humid subtropical climate	850 - 1000	Alfisol	24	20000	-	-	8.3	0.1	8.4	3	(Chirwa et al. 2004)
Tephrosia vogelii	Kenya	Af - Tropical rainforest climate	1400 -1800	Oxisol	6	NA	-	-	11.2	-	-	5	(Jama et al. 2008)
Tephrosia vogelii	Kenya				12		-	-	6	-	-	8	(Jama et al. 2008)
Tephrosia vogelii	Kenya				18		-	-	6.8	-	-	6	(Jama et al. 2008)
Sesbania sesban	Kenya -Kitalale-	Cfb - Subtropical highland climate	1200 - 1300	Oxisol	34	8300	5	1.9	6.9	1.5	8.4	4	Primary data from this study
Sesbania sesban	Kenya -Siaya-	Af - Tropical rainforest climate	1000 - 1250	Alfisol	34	8200	2.5	1.1	3.6	0.8	4.4	6	Primary data from this study
**Quartile 1**					***18***		***5***	***1.9***	***4.5***	***1.1***	***7.7***		
**Median**					***24***		***7.8***	***4.5***	***6.9***	***1.8***	***8.5***		
**Quartile 3**					***24***		***7***	***6.5***	***10.9***	***4.9***	***13.3***		

Appendix S4. Compilation of assumptions

Table S4.1. Assumptions used to dimension the integrated agroforestry-bioenergy system and quantify its potential outcomes.

**Assumption**	**Quantity**	**Unit**
General		
Farm size	1	ha
Area under tree biomass production	0.2	ha farm^{-1}
Fallow period	2	year
Cooking frequency	2	cooking occasions day^{-1}
Electricity use	500	kWh year^{-1} household^{-1}
	1200	kWh year^{-1} per small business or public service
Village size	50	households
	3	small businesses or public services
Household cooking with produced branches		
Fuel moisture content when used	15	% wet mass fraction
Fuel consumption in a rocket stove	0.8	kg fuel cooking occasion^{-1}
Fuel consumption natural draft gasifier	1	kg fuel cooking occasion^{-1}
Biochar produced in a natural draft gasifier	15	% input fuel mass
Electricity generation from the produced logs in CHP gasification plants		
Fuel moisture content when used	10	%
Electric power	40	kW
Thermal (heat) power	79	kW
Fuel consumption	38	kg h^{-1}
Biochar production	2.7	kg h^{-1}

Appendix S5. Data compilation for commercially available small-scale combined heat and power plants

Table S5.1. Technical specification of commercially available small-scale (≤ 50 kW electric power) combined heat and power (CHP) gasification plants. Fuel consumption is expressed on wet-basis.

Name	Manufacturer	Electric Power (kW)	Heat Power (kW)	Fuel consumption (kg/h)	Biochar production (% of input)	Link
V4.50	Burkhardt	50	110	40	-	https://burkhardt-gruppe.de/en/power-engineering/heat-and-power-from-wood/wood-gas-generator/holzvergaser-v-4-50/
ECO20X	CMD	20	40	24	-	https://eco20cmd.com/en/eco20x/why-choose-eco20x-for-producing-energy/
CHiP50	ESPE	49	110	49	Max 5 (Ash)	*https://www.espegroup.com//app/uploads/2021/02/Datasheet_CHiP50_ENG.pdf*
Fröling	CHP46	46	95	35	-	https://www.froeling.com/en-gb/products/heat-and-electricity/chp/
Fröling	CHP50	50	105	37	-	
Glock Ecoenergy	GG-GHP 1.7	18	44	18	-	https://www.glock-ecoenergy.com/en/GG-CHP
Glock Ecoenergy	GG-GHP 2.7	50	110	50	-	
Holz Kraft	HKA 35	35	79.5	31.5	-	https://www.holz-kraft.com/en/products/hka-35-45-49.html
Holz Kraft	HKA 45	45	102.2	40.5	-	
Holz Kraft	HKA 49	49	111.3	44.1	-	
Walter	Volter	50	120	*		https://volter.fi/en/products/walter-powerplant/
HKW50	Lipro Energy	50	97	37.5	-	https://www.lipro-energy.de/en/products-and-services/lipro-wood-gasification-power-plant/
PowerSkid 19+	Reset	19	28	22.8	7	https://www.reset-energy.com/en/syngasmart/
PowerSkid 35+	Reset	35	51	42	6.9	
PowerSkid 50+	Reset	50	73	60	7	
PP30	All Power Labs	25	50	25	-	https://www.allpowerlabs.com/wp-content/uploads/2019/08/PP30OneSheet2_1_23-2.pdf
**Average**		**40**	**79**	**38**	**7**	

* Walter by Volter uses “ca. 5.6 loose cubic meters/24 h”

References

Chikowo, R., P. Mapfumo, P. Nyamugafata, and K. E. Giller. 2004. Woody legume fallow productivity, biological N2-fixation and residual benefits to two successive maize crops in Zimbabwe. Plant and Soil **262**:303-315.

Chirwa, T. S., P. L. Mafongoya, D. N. M. Mbewe, and B. H. Chishala. 2004. Changes in soil properties and their effects on maize productivity following Sesbania sesban and Cajanus cajan improved fallow systems in eastern Zambia. Biology and fertility of soils **40**:20-27.

Clifford, D., N. Cressie, J. R. England, S. H. Roxburgh, and K. I. Paul. 2013. Correction factors for unbiased, efficient estimation and prediction of biomass from log–log allometric models. Forest ecology and management **310**:375-381.

Finney, D. J. 1941. On the Distribution of a Variate Whose Logarithm is Normally Distributed. Supplement to the Journal of the Royal Statistical Society **7**:155-161.

Jama, B. A., J. K. Mutegi, and A. N. Njui. 2008. Potential of improved fallows to increase household and regional fuelwood supply: evidence from western Kenya. Agroforestry Systems **73**:155-166.

Kottek, M. G., Jürgen; Beck, Christoph; Rudolf, Bruno; Rubel, Franz. 2006. World Map of the Köppen-Geiger climate classification updated. Meteorologische Zeitschrift **15**:259 - 263.

Mafongoya, P. L., and B. H. Dzowela. 1999. Biomass production of tree fallows and their residual effect on maize in Zimbabwe. Agroforestry Systems **47**:139-151.

Shen, H., and Z. Zhu. 2008. Efficient mean estimation in log-normal linear models. Journal of Statistical Planning and Inference **138**:552-567.

Ståhl, L., G. Nyberg, P. Högberg, and R. J. Buresh. 2002. Effects of planted tree fallows on soil nitrogen dynamics, above-ground and root biomass, N2-fixation and subsequent maize crop productivity in Kenya. Plant and Soil **243**:103-117.

Kottek, M., J. Grieser, C. Beck, B. Rudolf, and F. Rubel. 2006. World Map of the Köppen-Geiger climate classification updated. Meteorologische Zeitschrift **15**:259 - 263.

USDA–NRCS. 1999. Soil taxonomy—A Basic System of Soil Classification for Making and Interpreting Soil Surveys. Number 436, United States Department of Agriculture Natural Resources Conservation Service.
